# Endogenous N-acetylaspartylglutamate (NAAG) inhibits synaptic plasticity/transmission in the amygdala in a mouse inflammatory pain model

**DOI:** 10.1186/1744-8069-6-60

**Published:** 2010-09-22

**Authors:** Mary O Adedoyin, Stefano Vicini, Joseph H Neale

**Affiliations:** 1 Department of Biology, Georgetown University, Biology Reiss Building 37th and O St. NW, Washington, D.C. 20057 USA; 2 Interdisciplinary Program in Neuroscience, Georgetown University School of Medicine Med-Dent Building, NE 112, Washington, D.C. 20057 USA; 3 Department of Physiology and Biophysics Georgetown University School of Medicine Basics Science Building, Room 225, 3900 Reservoir Road, NW Washington, D.C. 20007 USA

## Abstract

**Background:**

The peptide neurotransmitter N-acetylaspartylglutamate (NAAG) is widely expressed throughout the vertebrate nervous system, including the pain processing neuraxis. Inhibitors of NAAG peptidases are analgesic in animal models of pain. However, the brain regions involved in NAAG's analgesic action have not been rigorously defined. Group II metabotropic glutamate receptors (mGluR2/3) play a role in pain processing in the laterocapsular part of the central nucleus of the amygdala (CeLC). Given the high concentration of NAAG in the amygdala and its activation of group II mGluRs (mGluR_3 _> mGluR_2_), this study was undertaken using the mouse formalin model of inflammatory pain to test the hypothesis that NAAG influences pain processing in the amygdala. Evoked excitatory postsynaptic currents (eEPSCs) were studied in neurons in the CeLC of mouse brain slices following stimulation of the spinoparabrachial amygdaloid afferents.

**Results:**

Application of a NAAG peptidase inhibitor, ZJ43, dose dependently inhibited the amplitude of the eEPSCs by up to 50% in control CeLC demonstrating the role of NAAG in regulation of excitatory transmission at this synapse. A group II mGluR agonist (SLx-3095-1) similarly inhibited eEPSC amplitude by about 30%. Both effects were blocked by the group II mGluR antagonist LY341495. ZJ43 was much less effective than SLx in reducing eEPSCs 24 hours post inflammation suggesting an inflammation induced reduction in NAAG release or an increase in the ratio of mGluR_2 _to mGluR_3 _expression. Systemic injection of ZJ43 proximal to the time of inflammation blocked peripheral inflammation-induced increases in synaptic transmission of this pathway 24 hrs later and blocked the induction of mechanical allodynia that developed by this time point.

**Conclusions:**

The main finding of this study is that NAAG and NAAG peptidase inhibition reduce excitatory neurotransmission and inflammation-induced plasticity at the spinoparabrachial synapse within the pain processing pathway of the central amygdaloid nucleus.

## Background

The peptide neurotransmitter N-acetylaspartylglutamate (NAAG) has a positive role in animal models of traumatic brain injury, stroke, schizophrenia, inflammatory pain and peripheral neuropathy (reviewed in [1,2]). NAAG is widely distributed in the brain and spinal cord, including the ascending and descending pain pathways [3,4]. NAAG activates group II metabotropic glutamate receptors (mGluR_3 _> mGluR_2_) [5-7]. Two enzymes, glutamate carboxypeptidase II and III (GCPII and GCPIII), that inactivate synaptically released NAAG have been cloned and characterized [8-10] and a series of NAAG peptidase inhibitors have been developed [2,11]. These inhibitors have been used to define the effects of synaptically released NAAG in vivo.

Systemic, local and central applications of the NAAG peptidase inhibitors are analgesic in inflammatory and neuropathic pain models, an effect that is reversed by systemic administration of the group II mGluR antagonist, LY341495 [12-16]. It is hypothesized that NAAG exerts its analgesic effects by reducing glutamate release via the presynaptic group II mGluRs [1]. NAAG peptidase inhibition reduced synaptic release of glutamate at an identified synapse in the hippocampus, consistent with a study of the actions of NAAG in cell culture [17,18]. However, there have been no direct demonstrations of the actions of endogenous NAAG at other identified synapses, including those in the pain processing pathway.

The amygdala is involved in affective processing of sensory information including pain-related responses [19-22]. The central nucleus (CeA) is the main output of the multinucleated amygdaloid complex; its connections make it critical for expression of pain-related responses [19,21,23,24]. A glutamatergic synaptic pathway in the laterocapsular part of the central nucleus amygdala (CeLC) is involved in inflammatory pain processing [25]. Activation of the group II mGluRs significantly inhibited the evoked excitatory postsynaptic current (eEPSCs) in the CeLC in the rat arthritic model of inflammatory pain [26,27]. Given the expression of NAAG and NAAG peptidase activity in the amygdala [28-30], we speculated that NAAG activation of presynaptic group II receptors in the CeLC plays a role in regulating transmitter release and that elevation of synaptic levels of NAAG influences processing of inflammatory pain signals [1].

The NAAG peptidase inhibitor, ZJ43, was used to define the peptide's role in the spinoparabrachial amygdaloid afferent synapses in the CeA in brain slices from mice prior to and at different intervals after induction of footpad inflammation.

## Results

### Prolonged nociceptive behaviors in formalin mice model

#### Thermal hypersensitivity in formalin model

**Figure 1:**
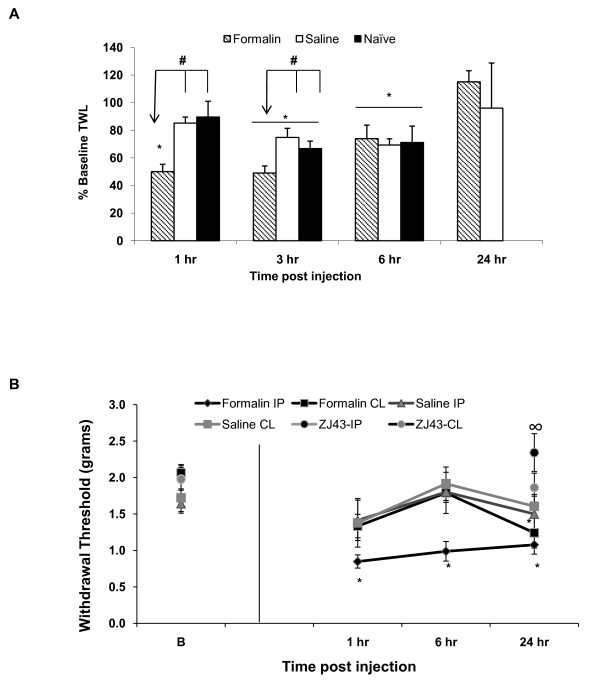
**Prolonged nociceptive behaviors in formalin mice model**. (**A**) Baseline thermal withdrawal latency (TWL) was determined for each mouse prior to treatment. This value was used to establish 100% baseline for that subject. Control groups (saline and naïve) mice showed habituation when retested at 3 and 6 hours. Thermal hypersensitivity (lower TWL relative to baseline as 100%) is observed in the formalin treated mice (n = 15) compared to the control groups (saline-treated n = 11; naïve n = 10) at 1 and 3 but not 6 hours post injection. The formalin treated group TWL was significantly different at 1 hr compared to naïve (p < 0.001) and (p < 0.005) saline groups; at 3 hr compared to saline (p < 0.02) and naïve (p < 0.05) groups. TWL returns to baseline values at 24 hours in the treated groups. #p ≤ 0.05 across group comparison for TWL values by two way RM-ANOVA followed by pairwise Tukey t-test post hoc analysis. (**B**) Mechanical allodynia, measures as paw withdrawal in response to force of probe in grams, was assessed in the injected hindpaws (ipsilateral = injected paw, IP) and opposite (contralateral, CL) hindpaws. There was a significant difference between the ipsilateral and contralateral footpad (p < 0.02) in the formalin mice. Ipsilateral hindpaw allodynia was significant at 1, 6 and 24 hours post-inflammation relative to the baseline values (n = 15). At 24 hours post-inflammation, the contralateral threshold was significantly (p < 0.05) lower compared to the baseline values in the contralateral footpad. ZJ43 (150 mg/kg; IP, n = 10) administered 30 prior to and 8 hours post formalin injection significantly reduced allodynia in the ipsilateral footpad (∞p ≤ 0.05, ZJ43 (formalin)-IP vs. Formalin-IP mechanical withdrawal threshold values at 24 hr post inflammation). The difference between ZJ43 (formalin)-CL and Formalin-CL did not reach statistical significance (p = 0.13). Two way RM-ANOVA was used followed by pairwise Tukey t-test post hoc analysis. Baseline (B) = probe force that induced paw withdrawal in mice prior to injection of formalin or saline. Data presented as mean ± SEM, *p ≤ 0.05 vs. baseline values; was used followed by pairwise Tukey t-test post hoc analysis.

Thermal withdrawal latency (TWL) response was repeatedly assessed in each mouse using the Hargreaves apparatus prior to and at 1, 3, 6 and 24 hours post injection into the footpad (saline- and formalin-injected groups). Thermal withdrawal latency (TWL) was significantly decreased at 1 and 3 hours post peripheral inflammation relative to saline treated (1 hr, p = 0.003; 3 hr, p = 0.02) or naïve (uninjected) mice (1 hr, p < 0.001; 3 hr, p = 0.04) (Figure 1A). The saline treated and naive mice habituated to the repeated testing and were no different from formalin treated mice at 6 hours (~70% baseline TWL for all groups). Both saline and formalin treated mice responses returned to baseline values by 24 hours.

#### Mechanical hypersensitivity in formalin model

Mechanical withdrawal threshold (allodynia) was repeatedly assessed in each mouse using the electronic von Frey anesthesiometer (EVF) system prior to and at 1, 6 and 24 hours post injection into the mouse's footpad (saline and formalin groups). The formalin treated mice exhibited prolonged mechanical allodynia in the injected left hindpaw that was significant at 1, 6 and 24 hours compared to baseline, while the contralateral hindpaw gave a significantly lower threshold response at 24 hours post injection compared to baseline but not at 1 and 6 hours. In contrast, the saline treated mice did not show any significant reduction in the threshold response (Figure 1B). The mechanical thresholds of the formalin injected hindpaws were significantly lower than those observed in the saline-injected animals at all time points post injection (p ≤ 0.05).

#### ZJ43 treatment prevents the development of mechanical allodynia in the formalin mice model

After baseline trials, two doses of NAAG peptidase inhibitor ZJ43 (150 mg/kg) was administered intraperitoneally (I.P.) 30 minutes prior to and approximately 8 hours after formalin injection into the mouse's footpad. This treatment inhibited peptidase activity for no more than 16 hours but not at 24 hour testing time (see Methods). The mechanical withdrawal threshold was then assessed at 24 hours post formalin treatment. NAAG peptidase inhibition blocked the development of mechanical allodynia in the injected hindpaw while the effect of ZJ43 failed to reach significance (p = 0.13) in the contralateral hindpaws at the 24 hr time point (Figure 1B).

### Peripheral inflammation results in synaptic changes in the CeLC region of the formalin mice model

#### Enhanced synaptic transmission in brain slices from formalin-treated mice

**Figure 2:**
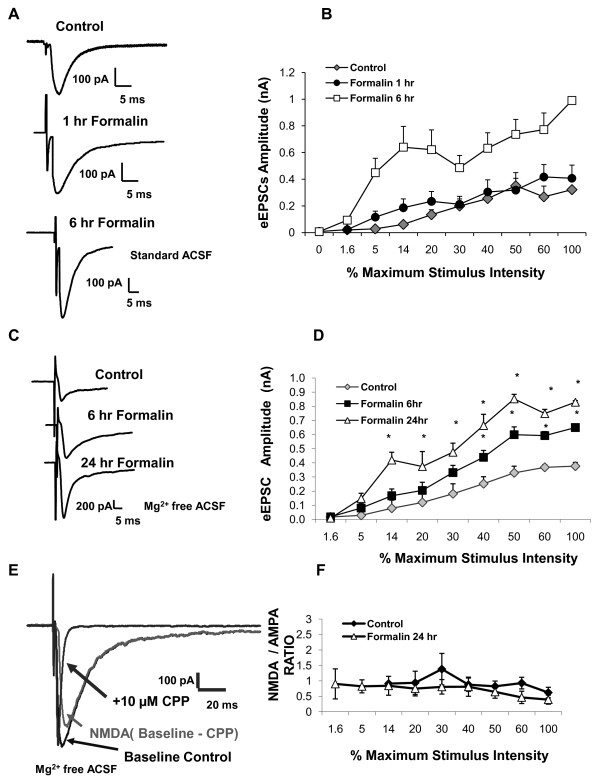
**Enhanced synaptic transmission in the CeLC nucleus of control and formalin brain slices**. Enhanced evoked current was observed in the central amygdala, laterocapsular part (CeLC) neurons 6 and 24 hours after peripheral inflammation. Input-output experiments were conducted to examine neurotransmission changes in the CeLC as a result of peripheral inflammation (QX-314 [5 mM] local anesthetic was included in the pipette solution). (**A**) Representative trace depicting evoked response at maximal stimulation intensity in cells from untreated control and formalin treated animals 1 and 6 hours post inflammation (standard ACSF).(**B**) An increase in current output was observed at 6 hours (n = 6) and not 1 hour (n = 9-13) in the CeLC post inflammation.(**C**) Representative trace depicting evoked response at maximal stimulation intensity in cells from untreated control and formalin treated animals 6 and 24 hours post inflammation (Mg^2+ ^Free ACSF). (**D) **There was a significant gain in the current output at 6 (n = 13) and 24 hours (n = 12) in the CeLC following inflammation. (**E**) Representative trace depicts how NMDA/AMPA ratio was obtained. NMDAR antagonist, CPP (10 μM), was used to determine the relative contribution of the NMDA and AMPA components of the evoked current response during input-output experiments (Mg^2+ ^Free ACSF). (**F**) NMDA/AMPA ratio was unchanged in the CeLC 24 hours after inflammation [control (n = 6), formalin 24 hours (n = 11)]. Data presented as mean ± SEM, *p ≤ 0.05 vs. control, Student's t-test.

To determine whether the behavioral changes observed are parallel with *in vitro *alterations in neuronal transmission in the laterocapsular part of the central nucleus of amygdala (CeLC), input-output experiments were conducted on mouse brain slices in standard ACSF at 1 and 6 hours post peripheral inflammation. The input output function of synaptic transmission was obtained by measuring the output current as a function of stimulus intensity. The magnitude of the evoked current was significantly increased 6 hours but not 1 hour after formalin injection relative to uninjected control experiments (Figure 2A &2B). When the input-output experiments were repeated in nominally Mg^2+ ^free ACSF recording solution at 6 and 24 hours after induction of peripheral inflammation, a significant gain in the current output was observed as a function of stimulus intensity (Figure 2C &2D).

#### NMDA/AMPA ratio remain unchanged in brain slices from formalin-treated mice

CPP (10 μM), a NMDA receptor antagonist, was used to pharmacologically evaluate the contribution of postsynaptic AMPA and NMDA receptors to the enhanced current observed in the input-output experiment at 24 hours post inflammation. The evoked currents in the presence of CPP were digitally subtracted offline from baseline control current to obtain the isolated NMDA component (Figure 2E). The observed current ranged from 10 to 800 pA but are not reported because the current output depended on the stimulus intensity. As a result, analysis of the NMDA/AMPA ratio was used because it allows for comparison between cells. The NMDA/AMPA ratio was not significantly changed 24 hours post inflammation compared to that obtained in uninjected control mice (Figure 2F).

#### Increased excitability of neurons in brain slices from formalin-treated mice

**Figure 3:**
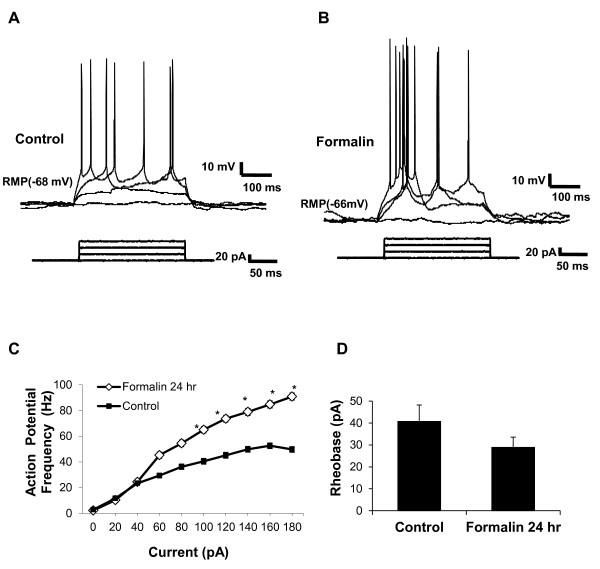
**Enhanced neuronal excitability in the CeLC neurons in brain slice of formalin-treated mice**. CeLC neurons in slices from untreated control and formalin treated mice were tested 24 hours after inflammation by injection with 20 pA current steps. Example of CeLC neuron firing patterns of untreated control (**A**), and formalin-treated mice (**B**) in response to the depolarizing current steps. (**C**) Average data showing increased action potential frequency in the CeLC neurons in brain slices 24 hours after inflammatory insult compared to untreated control [Formalin (n = 18), Control (n = 30)].(**D**) Average rheobase current in CeLC neurons in brain slices [Control (n = 28), Formalin (18)]. Data presented as mean ± SEM, *p ≤ 0.05 vs. control, Student's t-test. Recordings were in Mg^2+ ^free ACSF.

Following the observation of enhanced current output in the CeLC region, changes in the intrinsic firing properties of the neurons as a consequence of peripheral inflammation were investigated with current clamp recordings (Figure 3A control and 3B after inflammation). Neurons were injected with increasing depolarizing currents (20 pA steps, 200 ms) and the action potential firing rates were analyzed. Increased action potential firing rates were observed in the CeLC 24 hours after peripheral inflammation (Figure 3C). Mean input resistances were 197 ± 13.4 MΩ for control untreated mice (*n *= 31) and 225 ± 31.4 MΩ for formalin treated mice (*n *= 30) (p = 0.3, unpaired t-test). The rheobase current (see methods) for the formalin 24 hr group was lower but not significantly different from the uninjected control group (Control 41 ± 8 pA, n = 28; Formalin 29 ± 5 pA, n = 18 p = 0.2, unpaired t-test).

The increased firing rate and the enhanced evoked current suggest that in addition to an increase in transmitter release in the CeLC as a consequence of peripheral inflammation, there also were changes in the excitability of postsynaptic neurons (Figure 2D &3C).

### NAAG peptidase inhibition, NAAG and group II mGluR agonist affect synaptic transmission in the CeLC of control mouse

#### ZJ43 inhibits synaptic transmission in the CeLC

**Figure 4:**
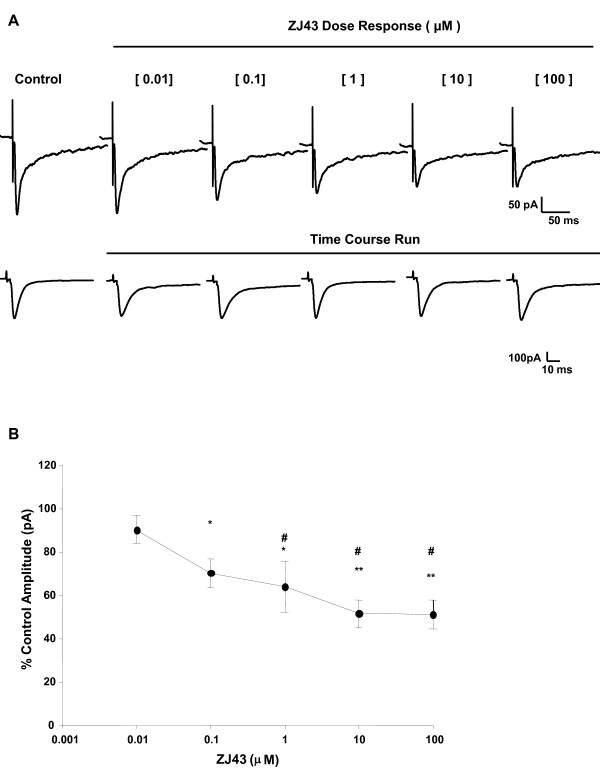
**Dose response effect of ZJ43, peptidase inhibitor, on evoked current in control mouse CeLC region**. (**A**) Representative evoked current traces shown in upper panel of ZJ43 at each concentration in a CeLC neuron; lower panel shows a representative cell current output traces over the same time duration as dose response experiments without a significant rundown in magnitude. (**B**) Average values for ZJ43 dose response effect on evoked excitatory currents in the CeLC of the untreated control mouse brain slice (n = 4). ZJ43 (10 μM) resulted in maximal (~50%) reduction in the amplitude of the evoked current. ZJ43's inhibitory effect at 100 μM (p < 0.01), 10 μM (p < 0.02) and 1 μM (p < 0.01) were significantly more potent compared to its effect at the minimum dose 10 nM. Data presented as mean ± SEM of percent control values, *p ≤ 0.05 vs. control, #p ≤ 0.05 vs. 10 nM ZJ43, one way RM-ANOVA followed by pairwise Tukey's t-test post hoc analysis. Recordings were in Mg^2+ ^free ACSF.

To define the role of endogenous NAAG on modulating glutamate release in the laterocapsular part of the amygdala (CeLC), the NAAG peptidase inhibitor ZJ43 was locally applied to the brain slice to prolong synaptic availability of NAAG following its release. ZJ43 treatment resulted in a maximal reduction in EPSC of 50% at 10 μM (Figure 4). Control experiments were conducted for the same duration as the dose response experiments; no significant change was observed in the amplitude of the evoked current over time (representative trace shown in lower panel, Figure 4A). Based on the dose response observations, 0.1 μM ZJ43 (approximately half maximal dose) was used to study the role of endogenous NAAG in the CeLC region.

**Figure 5:**
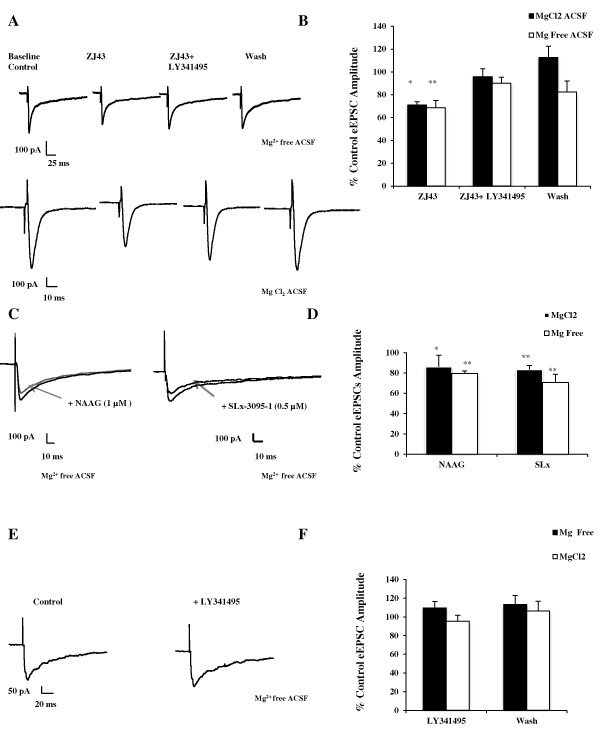
**Endogenous and applied NAAG inhibit synaptic transmission in the CeLC of the mouse amygdala**. (**A**) Representative current traces recorded from two neurons in the CeLC region of brain slices from untreated control mice following stimulation of the spinoparabrachial amygdaloid afferents in the absence and presence of MgCl_2 _in the ACSF. ZJ43 (0.1 μM) inhibits inactivation of endogenously released NAAG and significantly reduces the amplitude of the evoked excitatory current. This effect is reversed by the group II mGluR antagonist LY341495 (1 μM). (**B**) Bar graph showing average data in each recording condition (n = 7, Mg^2+ ^free ACSF; n = 6, standard ACSF). (**C**) Representative current traces from cells treated with NAAG (1 μM, n = 4) or group II mGluR agonist SLx-3095-1 (0.5 μM, n = 7). Both agonists inhibit the evoked current in the CeLC. (**D**) Bar graph showing average values from each drug treatment in the recording conditions. (**E **and **F**) Group II antagonist does not significantly affect evoked eEPSCs in control CeLC presented as mean ± SEM of percent control values. *p ≤ 0.05 vs. control, paired Student's t-test.

In brain slices obtained from untreated control mice, 0.1 μM ZJ43 resulted in a significant reduction (~30%) in the amplitude of the evoked current (Figure 5A, upper traces; Figure 5B). This indirect inhibitory effect of ZJ43 was blocked by co-application of LY341495 (1 μM), a competitive group II mGluRs antagonist. Similar effects of ZJ43 and LY341495 were obtained in the standard ACSF recording solution (Figure 5A, lower traces, and 5B).

To validate the effect of ZJ43 at this synapse, repurified NAAG (1 μM) and the group II mGluR agonist SLx-3095-1 (0.5 μM) were applied locally to the slice in nominally Mg^2+ ^free and standard ACSF recording conditions. NAAG and Slx-3095-1 caused a significant reduction in the amplitude of the evoked current (Figure 5C, left traces; 5D). To determine whether the group II mGluRs are tonically activated in the CeLC region of the uninjected control mice brain slice, the group II mGluR antagonist LY341495 (1 μM) was applied locally to the slice preparation in both Mg^2+ ^free and standard ACSF solutions. The antagonist had no significant effect on the amplitude of the evoked current in these control preparations in either recording solutions (Figure 5E and 5F; MgCl_2_, p = 0.76; Mg^2+ ^Free conditions p = 0.74) suggesting minimal activation of the group II mGluRs in the CeLC region of the brain slice at low stimulation frequency (0.1 Hz) in control mice.

**Figure 6:**
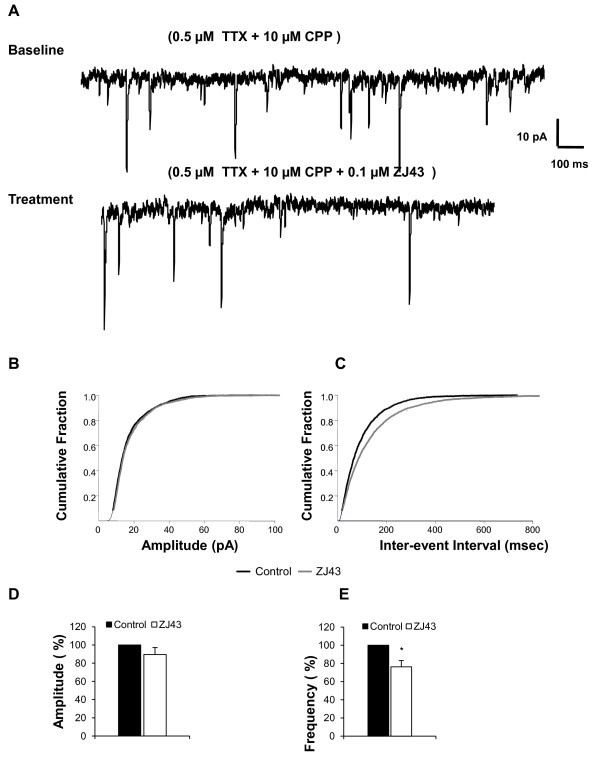
**Endogenous NAAG acts presynaptically to reduce the frequency of mEPSCs in the CeLC region**. Application of ZJ43 (0.1 μM) in the presence of TTX and CPP (to block action potentials and NMDA current respectively) in the CeLC region of the amygdala in slices from untreated control mice significantly reduced the frequency of miniature EPSC, while the amplitude of the mEPSCs was not significantly altered. **A**) Representative current traces in the absence and presence of ZJ43 in a neuron. (**B**) Cumulative amplitude curve in the presence and absence of ZJ43. (**C**) Cumulative inter-event interval curve depicting mEPSCs frequency rate in the presence and absence of ZJ43 in a neuron from a control untreated mouse brain slice. Bar graph showing % control of amplitude (p = 0.3, n = 9, ZJ43 treated versus control) (**D**) and frequency (**E**) values for cells (n = 9). Data presented as mean ± SEM, *p ≤ 0.05 vs. control, paired Student's t-test. Recordings were in Mg^2+ ^free ACSF.

To address NAAG's proposed presynaptic modulatory site of action, ZJ43's effect on miniature spontaneous EPSCs was determined in the presence of 0.5 μM TTX and 10 μM CPP to block sodium dependent action potentials and NMDA current respectively (Figure 6A). Application of 0.1 μM ZJ43 to the CeLC region significantly reduced the frequency of the mEPSCs (p ≤ 0.05, paired t-test, Figures 6C, E), while it had no significant effect on the amplitude of the mEPSCs (p = 0.32, paired t-test) (Figure 6B, D). The difference in paired pulse ratio (PPR) between control and ZJ43 treated cells was not significant (control 1.52 ± 0.13 vs. ZJ43 1.62 ± 0.18, p = 0.3 in paired t-test; wash 1.39 ± 0.10, n = 6).

### Endogenous NAAG effect in the CeLC following inflammation in the formalin mouse model

#### Endogenous NAAG inhibits synaptic transmission in brain slices from formalin-treated mice 6 and 24 hours after peripheral inflammation

**Figure 7:**
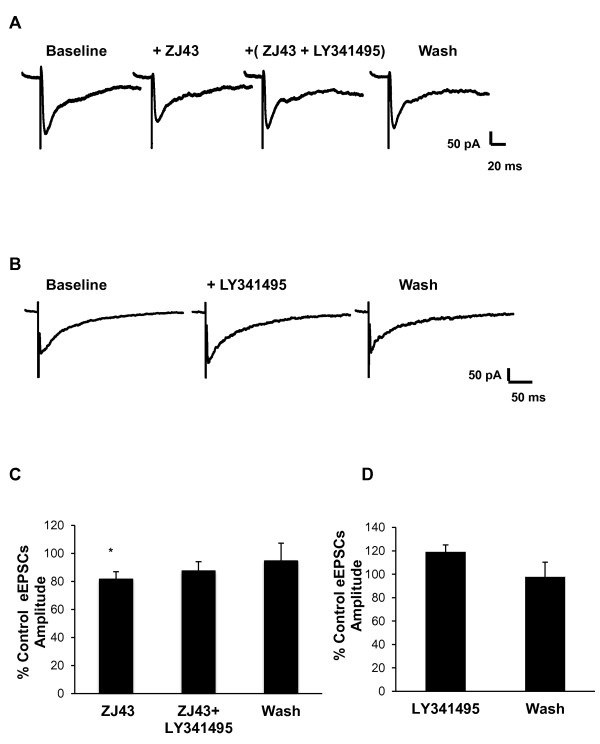
**ZJ43 inhibit the evoked current in CeLC neurons 6 hours after induction of peripheral inflammation**. The group II mGluR antagonist, LY341495 (1 μM), was used to define the level of tonic activation of the receptors as a consequence of peripheral inflammation. Representative traces: (**A**) ZJ43 (0.1 μM) significantly reduced eEPSCs in CeLC neurons; Average data showing (**D**) ZJ43 effects 6 hours post inflammation (n = 7). (**B**) In a separate set of cells, the direct effect of LY341495 was tested on the evoked current in the brain slice 6 hours post formalin treatment; (**C) **Average data showing LY341495 effect 6 hours post inflammation (p = 0.17, n = 7). Data presented as mean ± SEM percent control; *p ≤ 0.05 vs. control, paired Student's t-test. Recordings were in Mg^2+ ^free ACSF.

Given that synaptic plasticity was observed in the CeLC region as early as 6 hours post peripheral inflammation, the effects of ZJ43 (0.1 μM), NAAG (1 μM), and LY341495 (1 μM) were assessed on the amplitude of eEPSCs at this time point. ZJ43 significantly inhibited the amplitude of eEPSCs (Figures. 7A and 7C). Co-application of ZJ43 with LY341495 reversed the effect of the peptidase inhibitor's (Figure 7A). Exogenous NAAG tended to reduce the eEPSC amplitude to 76 ± 9.6% of control (p ≤ 0.05, n = 8 paired t-test, data not shown). In contrast to the control preparations, LY341495 alone appeared to increase eEPSC amplitude as might be expected if the antagonist blocked basal mGluR activation by endogenous NAAG, but this change did not reach statistical significance (Figure 7B and 7D, p = 0.15, n = 7, paired t-test).

**Figure 8:**
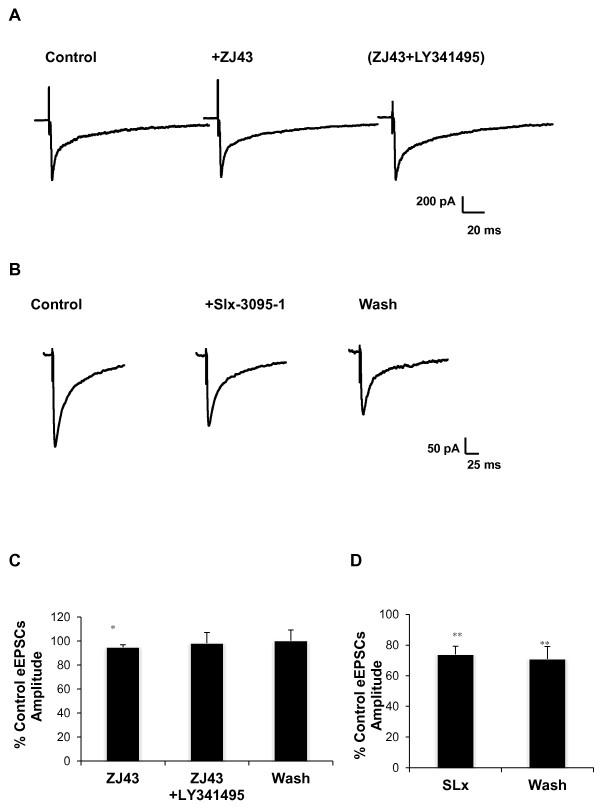
**The efficacy of ZJ43 and SLx-3095-1 in CeLC 24 hours post peripheral inflammation**. Whole cell recordings were conducted to investigate the inhibitory efficacy of ZJ43 (0.1 μM) and SLx-3095-1 (0.5 μM) in the CeLC 24 hours post peripheral inflammation. (**A**) Representative trace of the ZJ43 effect. (**C**) Average data for experiments shown in **A **in which ZJ43 alone had very little effect on eEPSCs (5% reduction, p < 0.01, n = 10, paired Student's t-test in contrast to its effect in controls (Figure 4). (**B**) SLx-3095-1 significantly reduced the amplitude of the eEPSCs in the CeLC region (n = 12), average data shown in (**D**). Data presented as mean ± SEM percent control; *p ≤ 0.05 vs. control, paired Student's t-test. Recordings were in Mg^2+ ^free ACSF.

In contrast to its efficacy in the amygdala from control mice (Figure 5B), ZJ43 (0.1 μM) had very little effect (~5%) on the evoked current at this synapse 24 hours after induction of inflammation (Figures 8A and 8C), while SLx-3095-1 again caused a substantial reduction in eEPSCs (Figures 8B and 8D).

#### IP injection of peptidase inhibitor, ZJ43, reduced inflammation-induced enhanced current output in the central nucleus of amygdala

**Figure 9:**
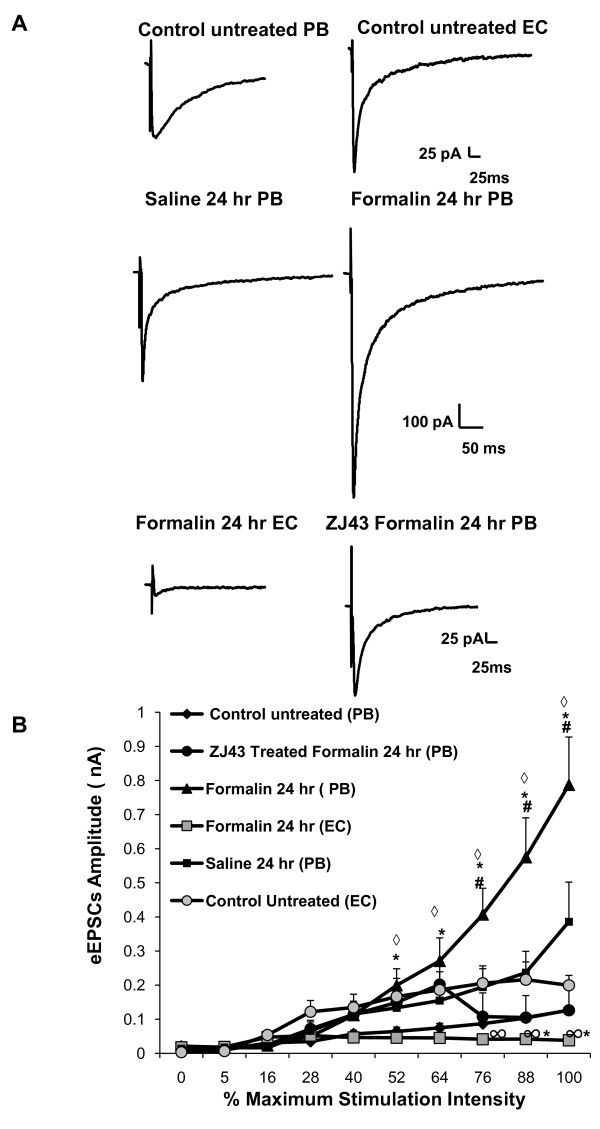
**PB-CeA and not EC-CeA synapse is activated in response to peripheral inflammation**. To investigate the specificity of the PB-CeA pathway output to the inflammation, the external capsule (EC) was stimulated while recordings were made from neurons present in the CeLC region of brain slices obtained from formalin treated animals. For these stimulus intensity experiments, the local anesthetic QX-314 (5 mM) was included in the pipette solution. (**A) **The enhanced current output induced by inflammation observed after stimulation of the PB afferents (middle panel, n = 5) did not occur when the EC was stimulated (lower panel, n = 5). Stimulation of the EC in the formalin brain slices yielded significantly lower current magnitude at the higher intensity compared to that obtained in untreated control brain slices (upper panel, n = 4). Injection (i.p.) of NAAG peptidase inhibitor ZJ43(150 mg/kg, n = 6, lower panel) 30 minutes before and 8 hours after formalin injection into the mouse's footpad significantly blocked the enhanced current output that would have occurred with stimulation of the PB afferents at the higher stimulus intensities in the formalin group (middle panel). Larger current output albeit not significant was observed in the CeLC region of brain slices obtained from the saline group (middle panel, n = 5) when compared to the untreated control group (upper panel, n = 5). (**B**) Average data showing the current output as a function of the stimulus intensity in all four animal groups (untreated control, saline treated, ZJ43- formalin treated, and formalin treated). For the treated groups the recordings were performed 24 hours post saline or formalin injection into the mouse's footpad. Afferents stimulation: (PB) indicates the parabrachial afferents, while (EC) indicates that the external capsule was stimulated. Data presented as mean ± SEM; *p ≤ 0.05 vs. control, #p ≤ 0.05 across group (Formalin 24 hr PB vs. ZJ43 Formalin 24 hr PB and Saline 24 hr PB), ◊p ≤ 0.05 across group (Formalin 24 hr PB vs. Formalin 24 hr EC),∞ p ≤ 0.05 across group (Formalin 24 hr EC vs. Control Untreated EC), unpaired Student's t-test. Recordings were in Mg^2+ ^free ACSF.

In this series of experiments (Figure 9), there again was a substantial increase in eEPSC amplitude with increasing stimulus intensity 24 hours after formalin injection. Two doses of the NAAG peptidase inhibitor ZJ43 (150 mg/kg, i.p.) were administered to the formalin mice group as in the behavioral studies (Figure 1). The peptidase inhibitor completely blocked the formalin-induced increase in the excitatory response at a time when there was little or no ongoing peptidase inhibition. This effect correlates with the findings shown in Figure 1B in which ZJ43 blocked the development of the mechanical allodynia that was observed 24 hours post inflammation.

To test the conclusion that these inflammation-induced changes in spinoparabrachial input to the central nucleus were specific to this pain pathway rather than generalized changes due to stress or other factors, recordings also were made following stimulation of the external capsule (EC) that carries cortical afferents input to the amygdala. No change in eEPSC amplitude was observed with increasing stimulus intensity in this circuit 24 hours after induction of inflammation (lower panel formalin 24 hr EC vs. middle panel formalin 24 hr PB, Figure 9A &9B). Instead a reduction in the current output was observed when compared to that obtained in control brain slices.

## Discussion

Peptidases that inactivate NAAG have been cloned, characterized and deleted in null mutant mice [9,10,31,32]. Inhibitors of these peptidases and agonists of NAAG's mGluRs have been studied as promising targets for drug development [1,2,11,33,34]. In contrast to other neurotransmitters, the actions of endogenously released NAAG have been difficult to rigorously characterize at identified synapses due to its conversion to glutamate by peptidases in the extrasynaptic space. Circumventing this, the application of NAAG peptidase inhibitors to slice preparations provides an opportunity to study the physiology of released peptide [17]. Importantly in this and previous pain studies, the effects of ZJ43 are blocked by the group II mGluR antagonist, LY341495, supporting the conclusion that the peptide was acting via these receptors. The data reported here represent the first to couple the synaptic action of NAAG to a discrete central sensory processing pathway in a basal state and as it changes in response to sensory input.

These data support the hypothesis that ZJ43-mediated increases in NAAG decreased glutamate release in the amygdala, although this remains to be directly demonstrated in this brain region via microdialysis studies. It is well established, however, that group II mGluRs located at presynaptic glutamatergic synapses decreased evoked transmitter release [35-40]. Consistent with this, we previously used ZJ43 to elevate extracellular NAAG levels and decrease glutamate flux from hippocampal neurons in vivo [41]. Important to interpreting studies using ZJ43, this peptidase inhibitor has no direct action on group II or other mGluRs [12,42]. An alternative interpretation of these data would be that the ZJ43-induced decrease in EPSCs resulted from decreased release of glutamate directly from NAAG hydrolysis. Militating against this interpretation are microdialysis data demonstrating that extracellular NAAG levels are insufficient to significantly contribute to basal or stimulated glutamate levels [41,43].

### Presynaptic action of endogenous NAAG

NAAG peptidase inhibition significantly reduced the frequency of the miniature excitatory currents in this pain processing synapse in the amygdala (Figure 6E) supporting a presynaptic action of NAAG. The consistent degree of inhibition of eEPSC amplitude by ZJ43 in the presence and absence of Mg^2+ ^in the bathing medium (Figure 5) provides further evidence for a presynaptic action inasmuch as a post-synaptic action would be unlikely to affect NMDA and AMPA currents equally. A structurally different NAAG peptidase inhibitor similarly reduced eEPSCs and increased the paired pulse ratio (PPR) at the mossy fiber-Ca3 synapse [17].

The efficacy of NAAG in reducing transmitter release supports the hypothesis that NAAG expands the dynamic range of synapses across the nervous system by dampening release of its different co-transmitters at high levels of excitation. This would be a particularly important concept under pathological states that are associated with excessive glutamate release [1,40,41,44]. The absence of an effect of the group II mGluR antagonist alone under the basal activity of the control CeLC (Figure 5E, F) would be predicted by such a dynamic range model as would the absence of neurological deficits in GCPII knock-out mice under unstressed conditions [31].

### Peripheral formalin-induced inflammation enhances current output in CeLC region

Elevated glutamatergic transmission in this spinoparabrachial amygdaloid pathway is involved in inflammatory pain processing [21]. The parabrachial nucleus (origin of PB-CeLC afferents) relays converging specific nociceptive information from the spinal lamina I to the forebrain. The spinal nociceptive inputs reach, both directly and via the parabrachial nucleus, all subcortical components of the central autonomic network, including the amygdala [45-48]. The amygdaloid nuclear complex provides emotional significance to sensory stimuli, including pain [25,49,50]. Synaptic plasticity observed in the CeLC region following formalin-induced inflammation (Figures 2 and 3) is consistent with data from the rat mono-arthritic [51,52] and spinal nerve ligation models [53]. A similar current increase was found in the cingulate cortex after footpad inflammation [54-56]. In addition, molecular changes, such as enhanced pERK levels, were reported in the right CeLC amygdala region of mouse brain slices as early as 3 hours after formalin injection into the left or right footpad [57].

The change in efficacy of ZJ43 from control to 24 hr post inflammation (Figure 8), while the group II agonist remained effective under both conditions, is consistent with either a decrease in release of NAAG at the 24 hr point or a change in the ratio of the group II receptors in favor of mGluR_2_.

In contrast to the change in the PB pathway to the central nucleus 24 hrs after formalin injection, the EC evoked EPSCs is substantially reduced (Figure 9A). While this may have been due to plasticity induced by decreased cortical input the amygdala following peripheral inflammation, there are insufficient published data on cortical input to the amygdala under these conditions to permit such speculation.

### Endogenous NAAG's role in the PB-CeA circuit

NAAG, NAAG peptidase activity and group II mGluRs are present in the pain processing pathway [28,37-39]. While the efficacy of NAAG peptidase inhibitors in modulating perception of inflammatory pain has been studied extensively [14-16,58], discrete sites in the pain pathway have not been explored systematically for the peptide's action. The observation that ZJ43-induced elevation of NAAG has a role at the PB-CeLC synapse in control mice supports the view that this is one site where systemic ZJ43 might act to reduce acute perception of inflammatory pain. In contrast, the substantially lower effect of ZJ43 24 hours after induction of inflammation argues that NAAG peptidase inhibition would have less influence on the PB-CeLC contributions to long term inflammatory pain perception. However, systemic injections of the peptidase inhibitor proximal to the time of in formalin treatment blocked the enhanced current output observed in the CeLC region associated with inflammatory input (Figure 9B) and mechanical allodynia in the injected hindpaw (Figure 1B) at the 24 hour time point. This result supports the view that early induction of analgesia by NAAG peptidase inhibition sufficiently suppresses activity in the pain pathways of the CeLC as to preclude the induction of this dramatic change in the circuit's excitatory transmission.

## Conclusions

**Figure 10:**
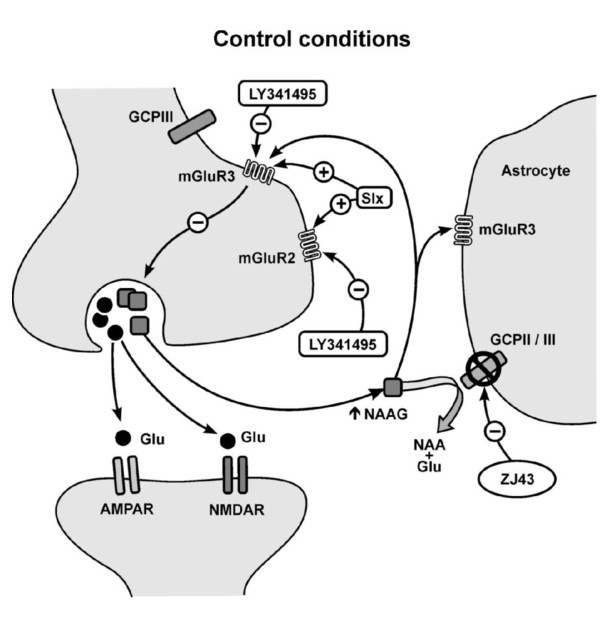
**Model of plasticity, mGluRs and NAAG in an inflammatory pain pathway of the mouse amygdala**. In the CeLC region of the amygdala of control mouse brains, ZJ43 inhibits CGPII (glial enzyme) and GCPIII (neuronal and glial enzyme). As a consequence, it effectively elevates the levels of synaptically released NAAG. The peptide acts relatively selectively at the mGluR_3 _to mediate its inhibition of glutamate release consistent with data on the actions of this peptide in other systems [1].

Data from this study support the following conclusions: (1) NAAG released at the spinoparabrachial amygdaloid synapse (PB-CeA) inhibits evoked release of its co-transmitter glutamate via presynaptic mGluRs (Figure 10); (2) over 24 hrs following induction of peripheral inflammation, PB-CeA afferents progressively exhibit enhanced evoked glutamate release and postsynaptic cells exhibit greater excitability; (3) this enhanced release is blocked by NAAG peptidase inhibition proximate to the time of the inflammatory insult; (4) within 24 hours after onset, peripheral inflammation induces a shift in the relative influence of NAAG peptidase inhibition on glutamate release at the PB-CeA synapse; (5) this inflammatory insult induces further physiologic plasticity: allodynia in the form of bilateral sensitivity to mechanical stimuli after 24 hours; (6) NAAG peptidase inhibition blocks the induction of this allodynia in the injected hindpaw.

## Methods

### Animals

Adult male C57BL/6 mice (postnatal day 17-25; Taconic, Germantown, NY) were housed with food and water available *ad libitum *in a temperature-controlled environment with light/dark cycle of 12:12 hour. All procedures were conducted in compliance with Georgetown University's Animal Care and Use regulations. All experiments were performed with the approval of the Georgetown University Animal Care and Use Committee. Effort was made to minimize the number of animals used to avoid unnecessary suffering.

### Formalin Test

Mice were anaesthetized via inhalation of 3% isoflurane and maintained with 1.5% isoflurane while the left footpad was injected. Mice recover with spontaneous activity promptly after removal of the anesthetic. Inflammation was induced by injecting 20 μL of 5% formalin (37% formaldehyde solution diluted in saline solution, 30 gauge needle) into the intraplantar surface of the mouse's hindpaw [59,60]. Control mice received 20 μL of saline solution (0.9%) into their hindpaw. Animals were monitored regularly after injections.

### Nociceptive testing: Thermal hyperalgesia

Hargreaves plantar apparatus (Ugo Basile, Camerio VA, Italy) was used to assess the thermal withdrawal latency (TWL) in the injected hindpaw [61]. Briefly, mice were allowed to acclimate (20 minutes) within a Plexiglas enclosure on a clear glass plate maintained at 30°C. When the mice were stationary, a mobile radiant heat source (i.e., high intensity projector lamp) located under the glass table was activated with a timer and focused onto the plantar surface of the left hindpaw. Thermal paw-withdrawal latency was determined by a motion detector that halted both lamp and timer when the paw was withdrawn. A maximal cut-off of 30 s was employed to prevent tissue damage. Paw withdrawal latencies were recorded automatically. Naïve (uninjected control), saline-treated, and formalin-treated animals were tested. Trials were repeated in the same animals prior to injection (baseline) and at 1, 3, 6 and 24 hours post-injection of either formalin or saline into the left footpad. Each trial was repeated twice at 3 minutes intervals, and the TWL is the average of the two responses. Behavioral response of flinching that may or may not be accompanied by licking of the heated paw was taken as a response. The heat stimulus gave a basal TWL of 7.1 ± 0.1 seconds.

### Nociceptive testing: Mechanical allodynia and threshold

For the evaluation of mechanical allodynia, mice were placed individually in clear Plexiglas enclosures on elevated mesh stand (large base 36" × 16") (IITC Life Science, Woodland hills, CA) to allow access to the surface of the hindpaw. The withdrawal threshold (WT) in grams was obtained by application of the rigid tip attached to the anesthesiometer transducer probe (2390 series Electronic von Frey Anesthiometer (EVF), Stoelting Co, Wood Dale, IL). The EVF system allows measurement and displays test readings upon reaction in grams based upon the amount of pressure applied. The EVF is zero calibrated before each use. When the mice were stationary, the stimuli were delivered from below, to the plantar surface of the hindpaw. The animals were acclimatized for 1 hour before behavioral testing and the mechanical allodynia was evaluated at several time-points (1, 6, and 24 hours). In order to determine the basal mechanical thresholds, all the groups were evaluated before the test procedures. Each trial was repeated twice at 5 minutes interval, the withdrawal threshold is an average of the two responses. Saline treated, formalin treated, and ZJ43- Formalin treated animals were tested.

### IP Injections

ZJ43 (150 mg/kg) was administered intraperitoneally (IP) 30 minutes prior to and approximately 8 hours after formalin injection into the mouse's left hind paw. Unpublished data from our lab indicate that a single i.p. injection of 150 mg/kg of ZJ43 inhibits NAAG peptidase inhibition for no more than 6 hours (Olszewski R, Ball S, Wegorzewska M, Lee M, Janczura K, Neale JH in preparation). As a result, in this dual injection paradigm there would be no significant peptidase inhibition ongoing at the time of behavioral testing or assessment of the brain slices at 24 hours post inflammation. The ZJ43 treated animals were divided into two groups: (1) used in electrophysiology experiments and (2) used in mechanical threshold (von Frey) experiments 24 hours post formalin injection.

### Electrophysiology

Coronal brain slices (300 μM) (Bregma -1.06 to -1.58 mm) containing the right central nucleus of amygdala, laterocapsular part (CeLC) were prepared using techniques that emulate those used in rats [52,62]. Briefly, mice (i.e. postnatal day 17-25, C57BL/6, Taconic Lab.) were decapitated, and the brain rapidly removed and placed in ice cold sucrose containing artificial cerebrospinal fluid (ACSF) buffer. No anesthesia was used prior to decapitation to avoid contamination of the brain tissue. Brain slices were then prepared using a micro slicer vibratome 3000 (Vibratome Inc, St Louis MO) and incubated at 32°C for 30 minutes in a sucrose based (ACSF) (mM): (NaCl 86, KCl 3, MgCl_2 _4, NaH_2_PO_4 _1, sucrose 75, glucose 25, CaCl_2 _1, NaHCO_3 _25). Slices were then maintained at 32°C for 30 minutes in standard ACSF of the following composition (mM): (NaCl 124, KCL 4.5, MgCl_2 _1, NaH_2_PO_4 _1, glucose 10, CaCl_2 _2, NaHCO_3 _26). The slice bathing medium was bubbled with O_2_/CO_2 _at a composition of 95% and 5%. After the incubation period, slices were transferred to the recording chamber and, unless stated otherwise, were perfused at 2 ml/min with a nominally Mg^2+ ^free extracellular solution (Mg^2+ ^was omitted from the standard ACSF). All experiments were performed at 30 ± 2°C in the presence of picrotoxin (30 μM, Sigma Aldrich St Louis, MO) and CGP52432 (10 μM, Tocris Bioscience Ellisville, MO) to block GABAergic currents. D-serine a co-agonist at glycine site on NMDA receptor (10 μM, Sigma) was included in nominal Mg^2+ ^free extracellular solution. Coronal brain slices were obtained from untreated control mice, saline treated mice (24 hours post injection), ZJ43 treated formalin mice, and formalin-treated mice at 1, 6 and 24 hours post footpad injection. Using video as a visual guide the stimulating electrode was positioned on the fibers dorsomedial to the CeA and ventral to but outside the caudaputamen, (see fig 8A in [63]). The spinoparabrachial amygdaloid (PB-CeA) afferents reaching the central nucleus of the amygdala were stimulated using a tungsten bipolar stimulating electrode at a frequency of 0.1 Hz. Stimulation of the afferents in the external capsule (EC), which sends cortical inputs that originate outside of the pain pathway (PB-CeA) to the amygdala was used as a control for input specificity. Recordings were made from neurons in the anterior part of the CeLC region.

Input-output experiments [63,64] were used to assess the synaptic strength response to incremental increases in stimulus intensity (0.08-5 mA) in the CeLC region and to compare changes in synaptic transmission between treatment groups. The same intensities were used for each animal group; the maximum stimulus intensity was that which failed to further increase the eEPSCs size. Current clamp experiments, with increasing depolarizing current injections of 20 pA steps were performed. Rheobase current was defined as the first current step, within a series of increasing 20 pA steps that elicited an action potential.

Electrodes were pulled in two stages on a vertical pipette puller from borosilicate glass capillaries (Wiretrol II, Drummond, Broomall, PA). The patch pipette solution composition (mM) was: (Kgluconate 145, EGTA 5, MgCl_2 _5, HEPES 10, ATP-Na 5, GTP-Na 0.2, and pH 7.2 with KOH). For the input-output experiments, the local anesthetic QX-314 (5 mM) was included in the pipette solution to block voltage gated Na^+ ^conductance. Typical pipette resistance was 5-7 MΩ. Whole-cell recordings at V_hold _= -60 mV were performed with a patch-clamp amplifier (Axopatch 200B, Axon Instrument, Foster City, CA) under video visual control with a Nikon Eclipse E600FN microscope (Nikon Japan).

### Drugs

Urea based glutamate carboxypeptidase (GCP) inhibitor N-[N-[(S)]-1, 3-dicarboxypropyl] carbamoyl]-L-leucine (ZJ43) was provided by Dr Alan Kozikowski at University of Illinois Chicago. At concentrations as high as 10 μM, ZJ43 fails to act as an agonist or antagonist at group I, II and III mGluRs [13]. The group II metabotropic glutamate receptor (mGluR) antagonist (LY341495), the GABA_B _antagonist (CGP 52432), the AMPA receptor antagonist [sodium-2, 3-dihydro-6-nitro-7-sulfamoyl-benzo[*f*]quinoxaline (NBQX)], NMDA receptor antagonist receptor antagonist [3-[(±)-2-carboxypiperazin-4-yl]-propyl-1-phosphonic acid (CPP)], and tetrodotoxin (TTX) were from Tocris Bioscience (Ellisville, MO). N-acetylaspartylglutamate (NAAG) from Tocris. We have long recognized that commercial NAAG is contaminated with 0.1-0.4% glutamate. As a result for more than a decade, we have routinely re-purified by ion exchange chromatography to remove traces of glutamate contamination (final glutamate levels less than 0.1%). The group II mGluR agonist SLx-3095-1 [see [16] was the generous gift of Alessandra Bartolozzi at Surface Logix, Inc. Boston, MA. SLx-3095-1 is the racemate (+/- isomer as HCl salt) of the highly selective group II mGluR agonist LY379268 (- isomer). Its synthesis is described as compound 9 in [65].

Stock solutions of TTX, NBQX, picrotoxin and CPP were dissolved in water. ZJ43, LY341495, and SLx-3095-1 were dissolved in saline. NAAG was dissolved in equimolar NaOH. CGP52432 was dissolved in dimethylsulfoxide (DMSO) at 0.01% final concentration.

### Drug application

The recording chamber was perfused continuously with nominal Mg^2+ ^free or standard ACSF in order to minimize spread of the drug beyond the application site. Drugs were diluted in the recording ACSF to obtain optimal concentrations. All drugs solutions were locally applied adjacent to the recording site via a Y-tube [66] for 5 minutes and modified for optimal solution exchange in brain slices [67]. AMPA receptor antagonist (NBQX, 5 μM) or NMDA receptor antagonist (CPP, 10 μM) were used at the end each experiment based on the type of current being measured to confirm the proper Y tube placement relative to the recording site.

### Data analysis

Spontaneous and miniature excitatory postsynaptic currents (sEPSCs and mEPSCs) were identified with semi-automated template based analysis software in Clampfit 9.2 or using a semi-automated threshold based mini detection software (Mini Analysis, Synaptosoft Inc., http://www.synaptosoft.com, Fort Lee, NJ) and visually confirmed. Event detection threshold was set at 5 times the root mean square level of baseline noise. mEPSC and sEPSC averages were based on ≥30 events in each cell studied and the decay kinetics were determined using exponential curve fitting. Evoked excitatory postsynaptic currents (eEPSCs) inhibited or enhanced by various pharmacological agents are represented as absolute magnitude and were determined by comparison of mean baseline before and immediately after drug application. Failures were excluded from evoked current analysis. The change in the peak of the Gaussians was calculated to determine the drug effect. Cells with > 20% change in access resistance were discarded. Off-line evoked current data analysis, curve fitting, and figure preparation was performed using Clampfit 9.2 (Molecular Devices, Inc. Sunnyvale CA) and Microsoft excel.

TWL and mechanical WT values were obtained from the digital readout of the Hargreaves plantar and EVF apparatus respectively.

Statistical significance was determined using a two-tailed Student's *t-test *unpaired when comparing two populations of cells, and paired when comparing conditions in the same cell population or animal group. In ZJ43 response experiments one way repeated measure-analysis of variance (RM-ANOVA) was followed by pairwise Tukey t-test post hoc analysis (Sigmaplot 11 software, http://www.sigmaplot.com).

In the von Frey experiments, differences between the three groups (saline, formalin and naïve) and changes over time were analyzed for ipsilateral and contralateral hindpaw with a two way RM-ANOVA, followed by Tukey post-hoc comparisons between groups. Significant changes post-injection were established by comparison with baseline values (pre-injection) using Student's t-test for paired data. In each group of the von Frey experiments, differences between contralateral and ipsilateral sides to injection were tested over time with a two way RM-ANOVA. Two way RM-ANOVA analysis was used to analysis changes in TWL over time. The Tukey t-test post-hoc analysis was used for multiple comparisons between groups. All data are presented as mean ± SEM. The significance levels were set at *p ≤ 0.05. In all figures, *p ≤ 0.05 and **p ≤ 0.005 compared to control values, ◊ or #*p *≤ 0.05 across groups. The term "control" when applied to brain slices or treatment groups refers to untreated mice.

## Competing interests

The authors declare that they have no competing interests.

## Authors' contributions

MOA carried out the behavioral assays and electrophysiological experiments. JHN and MOA conceived of the study and participated in its design and coordination. SV participated in the electrophysiology experiments design and coordination. MOA and JHN wrote the manuscript. All authors read and approved the final manuscript.
